# Using the Advocacy Coalition Framework to understand EU pharmaceutical policy

**DOI:** 10.1093/eurpub/cky153

**Published:** 2018-11-01

**Authors:** Eleanor Brooks

**Affiliations:** Global Health Policy Unit, University of Edinburgh, Edinburgh, UK

## Abstract

Models of interest group politics can help public health professionals (PHPs) to identify potential allies and establish mechanisms of sustainable political influence. This article focusses on a particular model, known as the Advocacy Coalition Framework (ACF), and its explanations of coalition behaviour, the role of scientific information and the ways in which coalitions can bring about policy change. The analysis illustrates the relevance of the ACF for public health by drawing on examples from the recent policy debate on direct-to-consumer advertising of prescription drugs (DTCA-PD) in the European Union (EU). It explores the strengths and weaknesses of the ACF in explaining why ‘control’ of particular governmental units was key to the anti-DTCA coalition success, how the evidence base was used strategically and why the pro-DTCA coalition ultimately failed in bringing about major policy change. The article aims to equip PHPs with a tool which can be used to understand and engage with the policy process. Moreover, in offering a more nuanced view of this process, a case is made for moving beyond traditional, linear conceptions of the policy process and engaging in further research which uses political science concepts to inform the study and practice of public health. The article concludes with a set of recommendations for practitioners and researchers, emphasizing the value of political science for the former and the need for the latter to reflect on the accessibility of policy studies for PHPs.

## Introduction

An understanding of interest groups and the coalitions that they form is vital to successful engagement in health policy processes. Models of interest group politics can help public health professionals (PHPs) to identify potential allies and establish mechanisms of sustainable political influence. This article focusses on a particular model, known as the Advocacy Coalition Framework (ACF), and its explanations of coalition behaviour, the role of scientific information and the ways in which coalitions can bring about policy change. The analysis illustrates the relevance of the ACF for public health by drawing on examples from the recent policy debate on direct-to-consumer advertising of prescription drugs (DTCA-PD) in the European Union (EU). The article aims to equip PHPs with a tool which can be used to understand and engage with the policy process. Moreover, in offering a more nuanced view of this process, a case is made for moving beyond traditional, linear conceptions of the policy process and engaging in further research which uses political science concepts to inform the study and practice of public health.

The ACF is a model designed to explain the behaviour of actors in debates on contentious public policy issues.^1^ Whilst there are plenty of others approaches which might be fruitfully used, the ACF is distinctive in being ‘…the closest thing to a general theory of policymaking’ and thus offering a comprehensive framework for thinking about political engagement.^2^ It understands the core of the policy-making process to be a competition between coalitions of actors with different belief systems.^2^ This article takes three core parts of the ACF—coalitions, belief systems and learning—and uses them to explain some of the main features of the DTCA-PD debate, before drawing some broader conclusions for political engagement by PHPs. The first section introduces the concept of coalitions and uses this to explain the range of actors involved in DTCA-PD policy-making and the importance of the relocation of responsibility for pharmaceutical policy within the European Commission in 2009. The second section discusses belief systems and how they shape the use of scientific information, illustrating how research was used to put DTCA-PD on the agenda and to maintain its salience. The third section explains the role of learning in facilitating policy change and analyzes the failure of the pro-DTCA-PD coalition to lift the ban on DTCA-PD, as well as the potential for a shift in EU regulation in future. The conclusion identifies implications for PHPs seeking to engage in health policy processes and presents recommendations for both practitioners and researchers.

The article draws on the author’s previous research on the DTCA-PD case.^3^ This research used documentary analysis, conducted while the author was resident at a non-governmental organization (NGO) closely involved in the development of the policy, as well as a review of the academic literature, to study the formulation of DTCA-PD policy. In the current article, this original data is analyzed alongside a review of the ACF literature. At specific points, reference is made to other literatures on, for instance, policy networks or the use of evidence in policy-making, though space precludes an in-depth review of all associated bodies of research.

The article illustrates the main tenets of the ACF using the case of DTCA-PD in the EU. From 2001 until 2011, a coalition of actors led by the pharmaceutical industry was engaged in a campaign to repeal (and later amend) the EU ban on DTCA-PD, so as to permit companies to provide information about their medicines directly to patients. The benefits of this, the pro-DTCA coalition argued, would be to educate consumers, improve doctor-patient dialogue and identify previously undiagnosed illnesses.^4^ Their efforts were challenged and eventually defeated by an opposing coalition, made up of actors concerned by evidence that DTCA-PD ‘medicalizes’ non-essential health issues and increases demand for drugs,^5–7^ as well as suggestions that ‘targeting of patients may be a prime objective’ of industry actors.^8^ The legislative proposal to weaken the DTCA-PD ban was withdrawn by the European Commission in 2014, having failed to gain agreement among EU policy-makers, and the debate has since shifted to one of ‘health literacy’ and broader information provision.^3^

## Coalitions, control and the role of ideas

The ACF understands change in public policy to be the result of interaction and competition between coalitions of actors within a particular policy area.^2^ These coalitions are made up of a broad range of interests and organizations from across local, national, regional and international levels of government, business and civil society groups, as well as journalists, researchers and policy analysts.^9^ In this way, the ACF has much in common with the literature on policy networks (see Löblová, this issue, and [2]) and epistemic communities^10^ in challenging the notion that policy, practitioner and research communities are distinct and internally homogenous.^11^ Mapping the actors involved in DTCA-PD policy from a coalitions perspective draws attention to contrasting positions held by different parts of the European Commission—the pro-DTCA leadership of Enterprise Commissioner, Erkki Liikanen, and the resistance of the Health Directorate, DG SANCO (now DG Santé)—and, perhaps less obviously, the disunity within civil society, between independent NGOs like Health Action International and their pharma-funded counterparts, such as the European Patients’ Forum.^5^^,^^12^ It also highlights the crucial roles played by actors such as EU journalist, Rory Watson, who published ongoing coverage in the *British Medical Journal*, co-director of *Social Audit Ltd*, Charles Medawar, who provided specialist policy analysis and academics such as Professor John Abraham, whose research on DTCA-PD was widely cited in the political debate. The ACF’s prioritization of ‘ideas’ is important here. Traditional, linear conceptions of policy-making assume an ordered process where evidence is considered at particular points and coordinated by a decision-making authority.^13^ However, as the ACF acknowledges, policy-making is no longer the purview of a closed group of individuals or institutions. Rather, ‘… actors may be influential because they articulate important ideas, not simply because they can exercise power’.^2^ Strong coalitions should therefore include a wide spectrum of actors, including those which may not have experience in political engagement.

The traditional policy-making institutions remain important. ‘In any intergovernmental system … different coalitions may be in control of various governmental units’ and a core objective of any coalition is ‘to … manipulate the assignment of program responsibilities so that the governmental units that it controls have the most authority’.^14^ Success, in this case, means ‘controlling’ crucial actors and positions. For example, the pro-DTCA coalition benefitted from influence over the Rapporteur in the relevant Parliament Committee, MEP Christopher Fjellner, who was committed to expanding the Swedish regulatory model, which permits industry communication with patients.^15^ The pro-DTCA coalition also ‘had control of’ the industry and enterprise directorate, DG ENTR.

The greater success was enjoyed, however, by the anti-DTCA coalition. In 2009 the pharmaceutical portfolio was reassigned from DG ENTR, where it had resided when the challenge to the DTCA-PD ban was launched, to DG SANCO, where it has remained (despite subsequent challenges) since. This reassignment was not an explicit element of the anti-DTCA coalition strategy, since DG SANCO’s weakness made it unlikely,^16^ but rather was an outcome of internal European Commission politics. DG ENTR was seen as unable to provide leadership in the H1N1 crisis; moreover, relocation would bring the Commission’s structure for pharmaceuticals in line with that of most member states.^17^ The ACF might define this shift as an ‘external system event’ or a change in a ‘long-term coalition opportunity structure’, both referring to features outside of the pharmaceutical policy sphere and the control of its actors. However, this sits uneasily with the reality that, in other circumstances, reassignment might have been a strong strategic objective for the anti-DTCA coalition and one which it might have had power to influence. Moreover, whilst the ACF recognizes the importance of ‘control’ of governmental units, it is less clear on how this interacts with other features of the system—changes in government, public opinion, etc.—as more recent literature acknowledges.^18^

## Belief systems and the role of technical information

In addition to challenging the structure of traditional, linear policy-making models, the ACF also challenges their logic. Refuting the notion that ‘healthy policy’ is the result of rational consideration of the evidence by committed political actors, the ACF holds that actors ‘… engage in politics to translate their beliefs, rather than their simple material interests, into action’.^2^ Since organizational and commercial interests are understood as beliefs, and beliefs can only be revised in particular, political circumstances, the ACF holds that evidence is a political tool that is unlikely to be used neutrally.^19^

This might sit uneasily in a sector where industry involvement in policy-making is more commonly understood as a conflict of interest than a divergence of beliefs but the ACF offers a valuable view on what role evidence can hope to play in changing the position of commercial actors and decision makers. It identifies three categories of belief.^14^ The first is ‘deep (normative) core’ beliefs, which can be thought of as an actor’s underlying personal philosophy, illustrated by their view on the relative value of freedom, power, health and knowledge, for instance. Pharmaceutical industry actors value ‘open markets, free enterprise and unrestrained exchange of information’,^4^ whilst patient organizations regard individual health and its protection as more important—this is a conflict in deep core beliefs. The second category of belief is the ‘near (policy) core’ belief, concerning issues like the appropriate balance between market and state activity and the desirability of participation in the policy process by experts. These are linked to the realization of deep core values in a particular policy sector. In extolling its value as a source of information about its medicines (a near core belief), the industry sought to realize its deep core commitment to freedom of information provision. Finally, there are ‘secondary’ beliefs. These resemble practical decisions about how to implement near core beliefs in a given policy issue, by establishing appropriate budgetary allocations and administrative procedures.^14^

Since change in deep core beliefs is ‘akin to religious conversion’^14^ and near core beliefs are unlikely to change within a single legislative cycle, secondary beliefs—those about the ‘routine delivery of specific policies’—are the most susceptible to change.^2^ This makes clear why health advocates struggled to change the views of their opponents in the DTCA-PD debate, and suggests that the pro-DTCA coalition was right to target incremental change in policy delivery, such as pilot projects to provide information on medicines for a specific list of diseases or a reframing of the debate to discuss ‘health literacy’.^3^ However, the ACF is less clear about how the profit-making interests of commercial actors should be understood. Its founding authors, in fact, are divided as to whether profit-making is the embodiment of a relatively fixed near-core belief—meaning that it will be difficult to convince the pharmaceutical industry that it should give up its entitlement to pursue profit by advertising its medicines—or a motive that means commercial actors are liable to change coalitions and beliefs frequently.^13^^,^^20^ This is an important weakness of the ACF and one which calls for further empirical investigation.

In addition to explaining actor behaviour, the ACF identifies a number of principles which govern the role of scientific information in the policy-making process. Here again, the ACF has much in common with the more recent literature on evidence in policy.^21^ The generation or gathering of evidence is prompted by a perceived threat or opportunity. For example, having noted the relaxing of DTCA-PD rules in the United States in the late 1900s, the pro-DTCA Transatlantic Business Alliance (TBA) quickly published a report listing the potential benefits of similar deregulation in the EU, pushing the issue onto the agenda (TBA quoted in [5,22]) Once the coalitions have adopted their positions, however, evidence is primarily used for advocacy purposes.^9^ The strategic value of scientific information is more important than the evidence it contains and partisan application is more common than apolitical consideration, even where knowledge is ‘co-produced’.^23^ The real value of the pro-DTCA coalition’s provision, inserted into Directive 2004/27/EC (Article 88a) and requiring a report on current DTCA practice within three years, was its role in ensuring that the issue would return to the agenda. The debate was reignited in 2007 and numerous consultations and studies quickly followed to support the position that ‘patient information’ was a topic requiring legislative action.^12^ Though only 7% of healthcare organizations, 11% of regulators and 0% of consumer and social insurance organizations responded positively to the idea of industry providing information to patients in a 2008 consultation,^24^ a proposal to this end was published nonetheless.^25^ In addition to supporting the ACF’s assumption that coalitions ‘… will resist information suggesting that their basic beliefs may be invalid or unattainable’,^14^ this is line with the broader literature on scientific information in policy and the conclusion that even evidence designed to inform policy-makers, like consultations, has primarily symbolic value as a ‘marker of good decision making’.^26^

## The role of learning in policy change

How then, in light of these features of the policy-making system, can PHPs hope to change policy? Since major policy change would require a shift in near core, and possibly even deep core, beliefs, this can only be brought about by an external event.^14^ For example, the election of a new government which favours DTCA-PD, or a health crisis linked to a lack of patient awareness about how a particular drug acts, could shift the balance of power. Here the ACF might offer some explanation of the failure of the pro-DTCA coalition to secure abolition of the EU ban. Whilst industry actors sought to harness the momentum from U.S. deregulation, this was not a significant enough shift in the external environment to undermine the EU’s commitment to consumer protection outright.

Thus, while coalitions must be ready to exploit such external events, minor policy change is perhaps a more predictable pursuit and a more achievable goal. The pro-DTCA coalition realized this early in the debate and sought to alter minor provisions of the EU regulation by adding amendments that allowed information provision in prescribed circumstances, such as for medicines to treat HIV/AIDS, asthma and diabetes.^12^ Similarly, the proposed Directive maintained the ban but allowed provision of ‘certain information’, understood to include that from scientific studies.^12^ Both of these attempts failed, but the role of learning in policy change, as understood by the ACF, facilitates a more nuanced analysis of the coalition’s strategy.

The early ACF literature identified learning as the key to minor policy change.^14^ Learning, where coalitions change their secondary beliefs in response to new information, has a specific meaning here.^27^ It is a political activity, where information and evidence are processed through the lens of deep held beliefs and often utilized for advocacy purposes, as explored above.^2^ As such, genuine learning will only take place in certain circumstances and its likelihood is determined by the degree of conflict between coalitions and the analytical tractability of the issue.^9^ The latter was less prominent in the DTCA-PD case—though the methodological rigour of many DTCA-PD studies is contested^28^—but can prove crucial in other areas of public health, such as calculations of alcohol-related harm in the minimum unit pricing debate, for instance. Where direct causality is difficult to assert and appropriate data sources and measurements cannot be agreed, coherent exchanges become difficult and the potential for learning is limited.

The greater the degree of conflict between coalitions, translating to the ‘depth’ of the disputed beliefs, the more information is produced and publicized, but the less receptive opposing actors become. On the surface, the DTCA-PD debate was about whether it is appropriate for the pharmaceutical industry to provide information about their products directly to consumers and how such provision should be regulated. At its core, however, was a conflict about the value of business and its promotion, relative to the value of consumer and health protection. DG ENTR, then, was unlikely to alter its view in response to evidence that DTCA-PD is designed to increase pharmaceutical sales^29^^,^^30^ and health advocates were unlikely to be moved by testimony of its virtues for informed choice.^31^^,^^32^ As such, the pro-DTCA coalition adopted the right strategy in seeking incremental change to the existing ban and, though the intensity of conflict meant that these efforts ultimately failed, some evidence of learning can be seen in the adoption of amendments (Directive 2004/27/EC Article 88a) and Council conclusions^33^ which called for ‘further reflection’ on the issue and the publication of more reports which would sustain attention.

## Conclusion

The ACF does not offer comprehensive explanation of the DTCA-PD debate in the EU but provides a valuable framework through which to analyze the strategies invoked by those involved. Moreover, by setting out some basic assumptions about the potential for learning between actors and the likely impact of scientific information, it makes clear the parameters and conditions of successful advocacy and policy influence. This concluding section identifies three key implications for PHPs and researchers.

1. Health actors must form broad coalitions and prepare to maintain involvement for a long time. Seek out non-traditional actors—journalists, researchers and those with less formal avenues of influence—as well as groups which are not yet mobilized but might be affected by the policy problem in future.^34^ Maintain these connections for the long run. Though ‘quick wins’ are appealing, processes of knowledge accumulation, learning and policy change take time and, as such, actors seeking influence should be prepared to engage in the policy cycle for a decade or more.^35^
2. Coalitions should be aware of belief systems and how they impact upon learning. Viewing policy debates through the lens of belief systems not only helps with the dissection and understanding of different views, but also enables clear articulation of one’s own views to others.^35^ Moreover, in being realistic about the chances of changing an opposing coalition’s position, an advocate is able to better frame and direct their expertise. As Jenkins-Smith and Sabatier^9^ warn,
  … it is in analytical debates characterized by high levels of conflict, over analytically intractable issues, and in open fora that analysis is most likely to be employed primarily as a political resource. Practitioners who expect their analysis to have an independent and influential role in shaping policy in contexts of this sort are likely to be met with disappointment.
3. Researchers must respond to calls for pluralistic and collaborative approaches to knowledge production at the intersection of public health and political science,^36^^,^^37^ particularly as concerns the policy-making process.^38^ This requires action on two fronts. PHPs and public health researchers should make use of the public policy and administration literature to move beyond linear conceptions of policy-making and acknowledge the difference between evidence-based medicine and evidence-based policy.^23^ Political science researchers, meanwhile, need to reflect upon the dilemmas that policy theory can present for PHPs, particularly assertions that influence requires persuasion and that hierarchies of evidence are not relevant for policy-making, which challenge core public health norms.^23^

## Funding

This open access Supplement received funding under an operating grant from the European Union’s Health Programme (2014–2020).

*Conflicts of interest*: None declared.

**Key points**

- The Advocacy Coalition Framework is one of several tools from political science which can help public health practitioners to engage with the policy process.
- Successful engagement requires identification of a broad and multi-disciplinary coalition of actors and a clear understanding of how beliefs shape learning and policy change
- Contrary to the dominant model of politics, scientific information is rarely considered apolitically—more commonly, it is used for advocacy purposes in support of a coalitions objectives.
- Political science research has great relevance for public health. Both practitioners and researchers should strive to ensure that synergies are exploited and knowledge is co-produced for maximum benefit.
